# SIUMB guidelines and recommendations for the correct use of ultrasound in the management of patients with focal liver disease

**DOI:** 10.1007/s40477-018-0343-0

**Published:** 2018-12-22

**Authors:** Gianpaolo Vidili, Ilario De Sio, Mirko D’Onofrio, Paoletta Mirk, Michele Bertolotto, Cosima Schiavone, The SIUMB experts committee, Esterita Accogli, Vincenzo Arienti, Raffaella Basilico, Luigi Bolondi, Paolo Busilacchi, Fabrizio Calliada, Vito Cantisani, Alder Casadei, Orlando Catalano, A. Maria de Gaetano, Giorgio de Stefano, Giulio Di Candio, Francesco Maria Drudi, Farina Roberto, Fornari Fabio, Giovanni Iannetti, Giovanni Maconi, M. Franca Meloni, Fabio Piscaglia, Maurizio Pompili, Gian Ludovico Rapaccini, Marcello Romano, Carla Serra, Luigi Solbiati, Maurizio Soresi, Stefania Speca, Luciano Tarantino, Massimo Valentino, Gianfranco Vallone, Angelo Assanti, Antonio Granata, Giulio Cocco

**Affiliations:** 1 Department of Medical, Surgical and Experimental Sciences, University of Sassari, Viale San Pietro 43b, 07100 Sassari, Italy; 2 Department of Hepatogastroenterology, Università degli Studi della Campania Luigi Vanvitelli, Naples, Italy; 3 Department of Radiology, G.B. Rossi Hospital, University of Verona, Verona, Italy; 4 Department of Radiology, Catholic University of the Sacred Heart- Fondazione Policlinico A. Gemelli, Rome, Italy; 5 Department of Radiology, University of Trieste, Ospedale di Cattinara, Trieste, Italy; 6 Unit of Ultrasound in Internal Medicine, Department of Medicine and Science of Aging, “G. d’Annunzio” University of Chieti, Chieti, Italy

**Keywords:** Contrast enhanced ultrasound, Hepatocellular carcinoma, Cholangiocellular carcinoma, Metastasis, Focal nodular hyperplasia, Hemangioma

## Abstract

The present document describes the SIUMB (Italian Society of Ultrasound in Medicine and Biology) guidelines for the use of ultrasound in the management of focal liver disease. The aim of the paper is to provide a clinical practice guideline for Italian physicians who are approaching the ultrasound study of a focal liver lesion. In particular, these guidelines provide simple indications, recommendations and general practice advices for the correct use of contrast-enhanced ultrasound (CEUS) in this scenario. They represent the SIUMB position of the ultrasound role in the diagnostic flow charts of the principal focal liver lesions, and are in agreement with other, previously published national and international guidelines.

## Preamble

This document represents the results of the Italian Society of Ultrasound in Medicine and Biology (SIUMB) guideline committee’s research concerning the use of conventional and contrast enhancement ultrasound in focal liver disease. In 2016, we started collecting data from literature (guidelines, scientific papers, and expert opinions) published over the past 10 years about the role of contrast-enhanced ultrasound (CEUS) in focal liver disease. Recommendations were formulated on the basis of the data analyzed. They were then evaluated by a panel of Italian physicians, experts in the use of ultrasound in focal liver disease. This “Consensus” was held in Rome, on 20 November 2017, during the last national conference. The results of the expert committee’s work were presented to SIUMB members on 21 November 2017, and the text, including recommendations, was then approved by the SIUMB executive bureau on 20 January 2018. This document is the summary of the SIUMB’s position regarding the use of contrast-enhanced ultrasound in focal liver disease. It aims to be a guideline to define the situations which are appropriate to propose a more sophisticated ultrasound imaging technique, such as contrast-enhanced ultrasound (CEUS), and when other imaging techniques need to be used.

## Motivations and methodology of the SIUMB guidelines concerning the use of ultrasound in focal liver lesions

The importance of ultrasound, and in particular the use of ultrasound contrast agents (UCAs), is well recognized in Italy, but we missed a guideline document developed by SIUMB. In the light of this lack, and on the strength of two decades’ experience using contrast-enhanced ultrasound, SIUMB set up a guidelines committee.

In the first meeting, held in Rome in September 2016, the authors carried out an analysis and selection of the already-published guidelines concerning the contributions of unenhanced and enhanced ultrasound to the diagnosis of focal liver lesions. The European Federation of Societies for Ultrasound in Medicine and Biology ( EFSUMB) guidelines, published for the first time in 2004, and updated in 2008, and the World Federation For Ultrasound in Medicine and Biology (WFUMB)–EFSUMB guidelines, in particular, were evaluated. The latter, published in 2013, represent the worldwide guidelines (published in *Ultrasound in Medicine and Biology* and in *Ultraschall in der Medizin*/*European Journal of Ultrasound*) [1–3]. All these documents show a deep analysis of the use of UCAs and its role and pattern in the characterization of each of the focal liver lesions.

After this analysis, we moved to evaluate the liver applications of CEUS in clinical scenarios, and its recognized role in the fields of hepatology and gastroenterology. This was a hot topic for Italian physicians, especially in the period between 2010 and 2013, because of the American Association for the Study of Liver Diseases (AASLD) decision not to consider the use of CEUS in the diagnostic flow chart of a new nodule developed in patients with chronic hepatitis or cirrhosis, after its role had already been recognized in previous guidelines published by the same society in 2005 [4, 5]. The reasons the AASLD gave for removing CEUS from the diagnostic flow chart of HCC were the possibility to misdiagnose cases of intrahepatic cholangiocarcinoma, although is very rare in cirrhotic liver (about 1–3%), and also the poor availability of such a diagnostic method in the United States of America [6]. The AASLD document was counterbalanced by a position paper developed by the Italian Association for the Study of the Liver (AISF) and published in 2013, in which CEUS was identified as a useful imaging modality in the diagnostic work-up of a new nodule developed in a cirrhotic liver [7]. This paper allowed Italian hepatologists to overcome the limit on the use of CEUS introduced in the AASLD guidelines published in 2010 and updated in 2011 [4, 5]. It happened after that, in previous years, other international societies had already adopted the use of contrast-enhanced ultrasound in hepatological clinical practice [8].

The American College of Gastroenterology (ACG) clinical guideline concerning the diagnosis and management of focal liver lesions, published in 2014, presented CEUS as an emerging modality that had some utility, especially in the diagnosis of benign lesions like hemangioma and focal nodular hyperplasia (FNH), but was not widely available in the United States. For this reason, the authors preferred to focus their attention on contrast-enhanced computed tomography (CECT) and contrast-enhanced magnetic resonance imaging (CEMRI) [9].

Finally, we evaluated two additional guideline documents. The first, published by the European Association For the Study of the Liver–European Organization for Research and Treatment of Cancer (EASL–EORTC) in 2012, which concerned the management of hepatocellular carcinoma, did not include any indications for the use of CEUS. The second, the European Association for the Study of the Liver (EASL) clinical practice guidelines on the management of benign liver tumors, published in 2016, acknowledged CEUS a diagnostic role in all benign lesions except for adenoma [10, 11].

To be complete and comprehensive, we evaluated two other documents after analyzing the international guidelines. These two documents were written in Italian. The first was published by the Ministry of Health in September 2008, as part of our national system of guidelines (SNLG). It was the first Italian document concerning the use of imaging in the diagnosis of focal liver disease [12]. The latter was the joint product of a number of different Italian scientific societies, namely: the Italian Association for the Study of the Liver Diseases (AISF); the Italian Association of Medical Oncology (AIOM); the Italian–International Hepato-Pancreato Biliary Association (IT-IHPBA); the Italian Society of Surgeons (SIC); the Italian Society of Medical and Interventional Radiology; and the Italian Society of Organs Transplantation (SITO), which wrote a document, published online in 2016, that included recommendations for the management of patients with hepatocellular carcinoma [13].

After the analysis of international and national guidelines, the second step was to evaluate the most important papers on the role of conventional and contrast-enhanced ultrasound in the management of patients with focal liver lesions. To do that, we carried out a bibliographic search by entering the following terms in PubMed: “liver cancers and ultrasound and contrast enhanced ultrasound.” The research was limited to the period between 2006 and 2016, and led to the identification of 899 articles. By activating filters for clinical trials, review and meta-analyses, we reduced these to 77 clinical trials, 132 reviews, 11 systematic reviews, and 4 meta-analyses [14–17]. We proceeded to filter these documents, only including: studies conducted on humans; studies in which the use of CEUS has been evaluated in terms of the identification and characterization of liver lesions, and the reporting data in terms of sensitivity/specificity or positive and negative predictive value (VPP-VPN); studies in which Sonovue (Bracco, Italy) was the only ultrasound contrast agent employed (we have excluded data related to the use of Sonazoid and Definity, because at the moment they are not available in our country); studies in which a qualitative evaluation of contrast medium has been performed (we have excluded studies in which quantitative assessments have been made with wash in/wash out time intensity curves, with or analysis of images using software such as Photoshop, etc.); studies in which there were at least 30 patients (with at least 10 benign liver lesions and 10 malign hepatic lesions); studies published in English; and studies in which the gold standard was the histological result, the CT and/or MRI diagnosis, or the clinical and radiological follow-up. In this first document, the SIUMB’s guidelines committee decided to focus mainly on the ultrasound diagnostic aspects of focal liver lesions, with no recommendations regarding the evaluation of tumor response after loco-regional treatment and systemic therapy. This topic will be addressed in a later document.

In drafting the final document, we decided to report the conclusions of the existing literature as recommendations, and to include the experts’ opinions on all the focal liver lesions presented.

The evidence for and strength of the recommendations were assessed according to the Grading of Recommendations Assessment, Development, and Evaluation (GRADE) system [18]. The strength of recommendations depends on the quality of the evidence. Each recommendation was graded as strong or weak. High-quality evidence corresponded to a strong recommendation, while a lack of or uncertain evidence resulted in a weaker recommendation.

The SIUMB’s experts committee voted on each of the statements. Each member of the committee had the ability to approve, disapprove or abstain from voting on a particular statement. A strong consensus was reached when there was agreement in > 95%, while broad consensus was achieved when > 80% of the experts agreed.

## General considerations about ultrasonography in focal liver lesions

### Introduction

Ultrasonography is usually the first imaging technique adopted by clinicians to study the liver. Its widespread use brought great advances in clinical hepatology. Before the advent of ultrasound and other imaging modalities like CT or MRI, it was almost impossible to discover focal liver lesions in asymptomatic patients, and the natural history of liver tumors was almost unknown to clinicians. Real-time ultrasonography is the most frequently used imaging procedure for primary diagnosis of focal liver lesions and in the survey of patients affected by chronic liver diseases and tumors of the gastrointestinal tract [19]. The evaluation of liver lesions has taken on great importance because of the increasing incidence of primary hepatic malignancies, especially hepatocellular carcinoma (HCC) and cholangiocarcinoma. In this arena, the first task of an ultrasound is to detect a focal liver mass, while the second is (possibly) to characterize it.

Generally, detection is made via B-mode ultrasound. In most cases, it is not possible to characterize and differentiate benign from malignant lesions using this mode, since it lacks specificity in characterizing focal liver lesions [20]. The characterization is a process that requires other technologies able to help a clinician in evaluating the vascular supply of the tumors. In the liver, such tumors are mostly hypervascular. In this sense, the advent of Doppler was an improvement. That technology made it possible to display the vascular abnormalities of a liver mass, but the availability of blood pool contrast agents for ultrasound, together with the development of harmonic imaging, has opened up new possibilities, both for the immediate characterization of any lesion detected in the liver, and for increasing the sensitivity of ultrasonography in the detection of liver metasteses [19–22].

### Ultrasound contrast agents and contrast-specific modes

The unique ultrasound contrast agent licensed in Italy and in Europe is Sonovue (Bracco, Milan, Italy). It consists of bubbles that have a flexible shell, allowing them to oscillate when insonated at a low mechanical index (MI). MI is a parameter that measures the acoustic power of the ultrasound beam and determines the harmonic echo frequencies, coming from the resonating bubbles, which can be constantly imaged in real time. The low mechanical index imaging technique has become the method of choice adopted by all the companies which produced ultrasound scanners. This method avoids the destruction of the bubbles and provides continuous dynamic imaging, which allows for the assessment of the arterial inflow, capillary distribution and venous outflow within a parenchymal organ. In turn, this enables the study of both macro- and microcirculation. Indeed, the size of the microbubbles allows free passage through the capillaries. Sonovue is distributed within the whole blood volume, but does not diffuse into the extracellular fluid space. A low mechanical index imaging technique using a second-generation ultrasound contrast agent has become the method of choice for the detection and characterization of focal liver lesions. The signal coming from the bubbles is detected by contrast-specific US modes, which work by erasing the linear ultrasound signal coming from the tissue and using the nonlinear responses from microbubbles to form images [2]. Each manufacturer has developed its own specific contrast mode, which effectively allows for tissue cancelation to generate almost pure microbubble images.

### Administration and dosing

Sonovue is administered by an intravenous bolus injection (preferably into an antecubital fossa vein), followed by a flush saline solution to push the agent into the central venous stream. The recommended dose for a single injection is half a vial, which amounts to 2.0–2.5 ml. However, the optimal dose for a particular clinical situation depends on the scanner technology used, and should be adjusted accordingly. The intravenous cannula should be at least 20 G in caliber, and the syringe should be connected directly or via the straight line of a T-connector. In this way, it is possible to avoid excessive mechanical stress during injection and prevent the destruction of the stable microbubbles [23].

### Safety of ultrasound contrast agents

UCAs are safe, with very rare side effects and an excellent tolerance in clinical practice. The incidence of severe hypersensitivity event is lower than that for CT contrast agents, and is comparable to that of MRIs. In abdominal applications, the incidence of life-threatening anaphylactoid reactions is 0.0001%, with no deaths in a series of > 23,000 patients [24]. Ultrasound contrast agents are not nephrotoxic, cardiotoxic or hepatoxic. Therefore, it is not necessary to perform renal function tests before doing them.

Although there is a theoretical possibility that the interaction of diagnostic ultrasound and UCA could produce bioeffects, there is no clinical evidence for adverse effects on human liver. For safety reasons, and to align with other guidelines, the SIUMB’s recommendations are:

- to ensure every patient provides informed, written consent before an examination is done;
- to work in a laboratory in which there are resuscitation facilities;
- to be careful with patients with a history of severe coronary disease; and
- to avoid using ultrasound contrast agents for 24 h before extracorporeal shockwave therapy.

### Examination technique and principle concepts in the interpretation of the images

Generally, contrast-enhanced ultrasound (CEUS) is performed after a gray-scale B-mode ultrasound, which represents the first step of the examination. It should be kept in mind that if the baseline ultrasound is suboptimal, CEUS may be unsatisfactory [3]. For best results, it is important to perform a high-quality un-enhanced ultrasound examination using tissue harmonic imaging and vascular Doppler technologies, to detect focal liver lesions and to find the best scan for the subsequent study with CEUS. Very small focal liver lesions, especially those located at the dome of the liver in sub-diaphragmatic segments, which occur mainly in patients affected by steatosis, may be difficult to identify in B-mode ultrasound, as well as with CEUS [3]. For these cases, we suggest intercostal scanning and positioning the patient in the left decubitus to improve the acoustic window. After the evaluation of the target mass, the next step is to move to contrast mode, maintaining the best detection scan. This switching causes the US scanner screen to split into two parts, one of which will be focused on the detection of the signal coming from the bubbles, while the other will be focused on the signal coming from the tissue. Some scanners allow for the possibility of merging both signals in a mixing mode with different colors. During contrast mode, which is an imaging modality with a low MI, the signal coming from the tissue is very useful and allows to for a guided scan that facilitates the characterization of the target.

The liver has a double blood supply, which consists of an arterial and a portal flow. 70% of the blood support arrives through the portal vein, while the remainder arrives via the hepatic artery. Considering that microbubbles trespass the capillary bed, we have to follow the dynamic change of the enhancement during the arterial, portal and parenchymal, or late, phase (Table 1). The arterial phase generally starts within 20 s after the injection of the contrast and lasts for 30–45 s, though the beginning depends on the circulatory status of the patient. The portal venous phase starts after 30–45 s and usually lasts until the 2-min mark, while the parenchymal phase starts after 2 min and is limited to 4–6 min.

**Table 1:** Contrast phases of the liver

Phase	Start (s)	End (s)
Arterial	10–20	25–35
Portal-venous phase	30–45	120
Late phase	> 120	Bubble disappearance (4–6 min)

The appearance of a lesion during the contrast mode should be described in terms of degree of the enhancement in all the phases. The term “enhancement” corresponds to the grade of perfusion with respect to the rest of the liver parenchyma. In this way, we can distinguish four perfusion patterns, which are, respectively, called hyperenhancement if there is hyperperfusion, hypoenhancement if the lesion is hypoperfused, and isoenhancement if it shows the same perfusion of the rest of the liver. Finally there are also not perfused lesions, in which there is no enhancement because there are no vessels inside.

The arterial phase is useful in the evaluation of the behaviors of the vascular arterial supply. In particular, it allows to focus on the type and timing of the filling (centripetal or centrifugal). Portal and late phases are more important, because in these phases, we can determine if the lesion is benign or malignant. A lesion that shows a decrease in the enhancement with a hypovascular aspect is typically malignant; while, if the lesions are iso- or hyperenhancing in the portal or late phases, they are typically benign. According to the literature, the smallest lesions that are detectable with CEUS range between 3 and 5 mm in diameter [25].

## Ultrasound aspects of focal liver lesions: un-enhanced and enhanced ultrasonography

### Introduction and clinical approach to the study of focal liver lesions

Before performing an ultrasound examination (B-mode and/or CEUS), it is also important to take the time to collect information concerning the clinical aspects of the patient’s life. This can help to produce a better diagnostic outcome. First of all, it is important to consider the age and the sex of the patient. In fact, the probability of finding a malignant lesion in a young person is lower than in an older patient. At the same time, the probability that particular kinds of focal liver lesions, like FNH or HCA, will be detected is higher in female patients. Another important issue is to know the clinical history of the patient. We can distinguish between three general scenarios:

- healthy patients with no history of cancer;
- patients with a history of cancer; and
- patients with a history of chronic hepatitis or cirrhosis.

Being aware of the clinical background can help us to better understand and forecast the type of lesion we are studying. For instance, oral contraceptive use in the absence of underlying liver disease could be suggestive of hepatocellular adenoma, while a new lesion identified in a patient with chronic hepatitis could be highly suggestive of HCC. The detection of a new lesion in a patient with a previous history of tumors could be suggestive of a secondary liver lesion.

### Ultrasound behaviours of different focal liver lesions: the results of the SIUMB expert committee

The following paragraphs describes the ultrasound aspects of focal liver lesions, putting in evidence unenhanced and enhanced ultrasound behaviors. Rare tumors are not discussed in this document, because at the moment there is not enough evidence for their detection with CEUS. There also indications and recommendations about following diagnostic flow charts in those cases in which ultrasound is not able to characterize a focal liver lesion. All the statements that follow represent a summary of the discussion which occurred during the SIUMB’s experts’ committee meeting, and constitute the Italian guidelines for approaching the study of a focal liver lesion.

#### Benign focal liver lesions

The benign focal liver lesions discussed in the following paragraphs are hepatic cyst, hydatid cyst, hemangioma, focal nodular hyperplasia, and adenoma. Focal fatty change was not considered properly a lesion, even though it can look like a mass, especially when hypoechoic and located in unusual sites. As an area of focal fatty infiltration or fatty sparing, it does not differ in vasculature from the surrounding liver tissue. In a contrast-enhanced ultrasound, it shows the same enhancement pattern as adjacent liver parenchyma in all the phases. Regenerative nodules are benign lesions developed in patients with cirrhosis, which generally show the same enhancement of the surrounding liver tissue, even though during the arterial phase, it could be possible to find a hypoenhanced aspect. The most important behavior of all the benign lesions in CEUS is that all of them show sustained enhancement in the portal and late phases. Their characterization depends on the type of enhancement during the arterial phase.

#### Hepatic cyst

Ultrasonography is highly accurate in the characterization of simple hepatic cysts, especially when typical features are present (anechoic appearance, thin walls, posterior acoustic enhancement and lateral acoustic shadows). Its sensitivity and specificity rates are higher than 90% [9, 10]. A simple, asymptomatic cyst should be managed conservatively (strong recommendation, moderate grade of evidence). Cystic lesions characterized by multiple and/or thick septa, wall irregularities, solid papillary projections, calcifications and/or daughter cysts should be investigated with color Doppler evaluation and contrast-enhanced imaging modalities. After CEUS, simple cysts (or those complicated by hemorrhage or infection) show no vascularization of contents and walls in all the vascular phases (strong recommendations, low grade of evidence). Strong consensus (35 approved/0 disapproved/0 abstained. 100%).

#### Parasitic cyst

The sensitivity of ultrasound for evaluation of Echinococcus is 90–95% [26]. The ultrasound behavior of this cyst depends on its evolutionary phase. At the beginning, it could appear as an anechoic, smooth, or round cyst, any of which can be difficult to distinguish from a benign cyst. With progression, the lesion may develop a thick and often calcified wall. Characteristic internal septa can be seen in the presence of daughter cysts. Ultrasound allows for a classification system that includes six classes (CL, CE 1,CE 2, CE 3, CE 4, and CE 5) divided into four groups (liquid cysts, activate parasitic cysts, transitional cysts, and inactive cysts) that correspond to the pathophysiology of the growth of the parasite [27]. The ultrasound behaviors of each class are reported in Table 2. The diagnosis of Echinococcus cysts requires the integration of ultrasound findings with serological tests, second-level imaging and the possible ultrasound-guided aspiration puncture of the endocystic fluid (strong recommendation, high grade of evidence). Strong consensus (35 approved/0 disapproved/0 abstained. 100%).

**Table 2:** Ultrasound behaviors of hydatid cysts

Type of cyst	Biological activity	Ultrasound features
CL	Undetermined	Cyst lesion (CL) with an anechoic content, in which the typical parasitic cyst wall is not detectable. This aspect could be present at the beginning of the infection by Echinococcus Granulosus. These ultrasound findings alone do not allow to perform the diagnosis of a parasitic cyst
CE-1	Active	Cyst lesion (CL) with an anechoic content, in which the typical cyst wall is detectable and the content could be characterized by echoes called hydatid sand
CE-2	Active	Multilocular cysts with septa, in which the typical cyst wall is visible
CE-3	Transitional	Cyst lesions, characterized by anechoic aspect and floating membranes, due to the separation of the laminated membranes from the cyst wall. These internal arrangements could modify the shape of the cyst by the decreasing of the internal pressure. In this stage the cyst could degenerate more or give daughter cysts. These ultrasound findings alone do not allow to perform the diagnosis of a parasitic cyst
CE-4	Inactive	Cyst with degenerative contents (hypoechoic or hyperechoic material). No daughter cysts inside
CE-5	Inactive	Calcified cysts with the involvement of the entire wall or part of it. These ultrasound findings alone do not allow to perform the diagnosis of a parasitic cyst

#### Hepatic abscess

Hepatic abscesses can appear in ultrasound as hypoechoic or hyperchoic lesions, depending on their size and the evolutionary status. Generally, however, they occur as hypoechoic lesions, with irregular walls and variable content (solid material and or gas) [28]. Their contrast-enhanced ultrasound behaviors depend on the state of the inflammation, which is an evolving process, and which progresses until liquefaction in mature abscesses [29]. The diagnosis of such abscesses is made by association between clinical signs and ultrasound imaging findings. In CEUS, abscesses show a peripheral rim enhancement during the arterial phase. This can persist in the portal phase, but usually decreases in the parenchymal phase. There is a lack of enhancement in the center of the lesion due to liquefaction. This pattern could be also observed in patients with infected granulomatous lesions. Ultrasound-guided puncture is useful for the characterization of the abscess, microbiological tests, and local treatment, that is, aspiration/drainage positioning. (Strong recommendations, moderate grade of evidence). Strong consensus (35 approved/0 disapproved/0 abstained. 100%).

#### Hepatic hemangioma

The typical ultrasound appearance of hepatic hemangioma is as a hyperechoic lesion with well-defined margins, but sometimes its aspect can be hypoechoic or inhomogeneous creating diagnostic doubts, especially in patients with oncological history or affected by chronic liver disease. The advent of contrast-enhanced ultrasound (CEUS) has improved both the sensitivity and specificity of US, reaching a high level of accuracy in the non-invasive diagnosis of the condition [9, 10, 30–32]. A hyperechoic lesion smaller than 3 cm (round shape, without a “halo sign” and with regular margins), detected in patients with normal liver ultrasound features and in the absence of a history of oncological disease, is probably a hemangioma and B mode ultrasound is sufficient for the diagnosis. In patients with hepatic steatosis, hemangioma may show an atypical hypoechoic appearance. In the presence of a large lesion, meanwhile, the ultrasound echostructure can be heterogeneous. Doppler evaluation is not useful for the diagnosis [10]. Typical hepatic hemangiomas with diameters < 3 cm do not require follow-up. When hemangioma is suspected, detected in patients with a history of cancer or affected by chronic liver disease, or when the lesion is bigger than 3 cm or shows atypical ultrasound features, it must be characterized with second-level imaging modalities, such as CEUS, CECT or CEMRI. The diagnosis of hemangioma with contrast-enhanced ultrasound is based on the typical peripheral globular enhancement pattern in the arterial phase, followed by centripetal filling in the portal and late phases [2, 3]. (Strong recommendations, moderate grade of evidence.) Strong consensus (35 approved/0 disapproved/0 abstained. 100%).

#### Focal nodular hyperplasia

The B-mode ultrasound behaviors of FNH are variable, but it is usually slightly hypoechoic or isoechoic, and very rarely hyperchoic. The central scar is visible on ultrasound in 20–30% of cases. After color Doppler evaluation, the lesion is hypervascularized, with hypertrophic vascular arteries that have the appearance of a spoked wheel. The lesion is characterized by vessels that rise from the center to the periphery of the nodule. Spectral Doppler analysis shows an arterial flow with a low resistive index.

When there are typical vascular characteristics, CEUS, CECT and CEMRI are able to reach a specificity level very close to 100%. In contrast-enhanced ultrasound, this lesion shows a rapid filling, starting from the center and moving out to the borders, with a centrifugal progression. In the arterial phase, it can take on a hypervascular aspect that can be maintained in all the phases, or it can show an isovascular appearance in the portal or late phase, comparable to that of hepatic parenchyma [9, 10, 32–36]. In the portal and late phases, the central scar, which has a hypovascular appearance, can be highlighted [37]. CEMRI shows a higher diagnostic accuracy rate for FNH. FNH does not typically require instrumental follow-up, except when it is associated with vascular malformative diseases [10]. (Strong recommendations, high grade of evidence.) Strong consensus (35 approved/0 disapproved/0 abstained. 100%).

#### Hepatic adenoma

The ultrasound aspect of the adenoma is variable and is conditioned by size, necrotic hemorrhagic evolution, and adipose content. Although study with color Doppler can document vascular signals, both arterial (with a reduced resistive index) and venous (both on the periphery and in the center), these aspects are not pathognomonic. CEUS is indicated for the differential diagnosis compared to the other focal liver lesions, but it is not accurate in confirming the diagnosis of adenoma or classifying the subtype [10, 38, 39]. (Strong recommendations, low grade of evidence.) Strong consensus (35 approved/0 disapproved/0 abstained. 100%).

#### Rare benign liver tumors

The available data on rare focal liver lesions do not allow for the formulation of diagnostic recommendations.

Strong consensus (35 approved/0 disapproved/0 abstained. 100%).

#### Malignant focal liver lesions

The malignant focal liver lesions discussed in the following subchapters are hepatocellular carcinoma, cholangiocellular carcinoma and metastasis, which represent the most common malignant masses. This document does not address other malignant lesions like lymphoma, epithelioid hemangioendothelioma or cystoadenocarcinoma, which are rare, and will be discussed elsewhere. Generally, all the malignant lesions show a hypoenhanced aspect in contrast-enhanced ultrasound during the portal or late phase, and the diagnostic accuracy of the method is very high [14–17].

#### Hepatocellular carcinoma

Ultrasonography is the imaging test universally proposed for the early detection of HCC in surveillance programs for cirrhotic patients. All patients with cirrhosis in Child–Pugh Turcotte (CPT) A or B, and those on the waiting list for liver transplantation, must be evaluated with periodic ultrasound [7]. The ultrasound surveillance program must also be extended to non-cirrhotic patients with chronic HCV infection or bridging fibrosis (Metavir F3), as well as to those patients being treated effectively with new directly active antiviral agents (DAA). An ultrasound surveillance program must be performed on HBV patients at high or intermediate risk. For Caucasian patients, the risk can be stratified according to the PAGE-B score, which is based on age, gender and platelets [40]. Ultrasonography should be performed every 6 months [7]. The examination should be carried out by experienced operators in dedicated centers and with high-level technological equipment. Each new nodule found in cirrhotic patients during a surveillance program, or in patients who do not adhere to surveillance programs, must be considered a potential HCC nodule. If the nodule is < 1 cm, the patient must be enrolled in a surveillance program, with ultrasound examinations at intervals of ≤ 4 months. If the lesion is ≥ 1 cm, the patient should be investigated with second-level imaging techniques [7]. Although conventional ultrasound and color Doppler can show suggestive features for the diagnosis of HCC (hypoechoic lesions with intra and perinodular arterial vascular signals and a high resistive index), the characterization of the nodule must be done using contrast-enhanced imaging modalities. CEUS can be used for the characterization of nodular lesions in patients with chronic liver disease. It allows for the characterization of the lesion as HCC when typical contrast enhancement pattern is present. HCC nodules generally show a hypervascular aspect during the arterial phase, followed by an isovascular and hypovascular aspect during the portal and parenchymal phases, respectively [2, 3, 38, 41]. CEUS must be performed by experienced operators, and the evaluation of the timing and the intensity of the washout is important [42]. In HCC nodules, the washout is frequently late (appearing after the first 60 s) and not very intense (the lesion does not appear markedly hypoechoic after 2–3 min). If the lesion shows washout within 60 s and appears markedly hypoechoic in portal phase, the possibility of malignant lesions other than HCC should be considered [41, 43]. The staging of HCC should be done with panoramic imaging modalities like CECT and/or CEMRI. CEUS does not have any kind of application in this field [7].

If imaging patterns are inconclusive after CEUS and CECT or CEMRI, biopsy should be performed. When portal vein thrombosis is present, CEUS allows the clinician to characterize the nature of thrombosis (neoplastic vs not neoplastic). In the case of neoplastic thrombosis, the thrombus appears “hypervascular” in the arterial phase, with washout in the portal and late phases. (Strong recommendations, high grade of evidence.) Strong consensus (35 approved/0 disapproved/0 abstained. 100%).

#### Cholangiocellular carcinoma

Intrahepatic cholangiocarcinoma (i-CCA) appears in conventional ultrasound as a nodular lesion that if < 3 cm, is most often hypoechoic, but the ultrasound aspect is completely indistinguishable from other liver lesions. The presence of segmental ectasia in the upstream biliary branches of the lesion can be suggestive, but is not specific to the diagnosis. Color Doppler has no role in the diagnosis of i-CCA.

In CEUS, the i-CCA behaves like a malignant lesion, characterized by a washout in the portal and late phases. Generally, the duration of the washout is shorter than that of HCC, and starts in the portal phase [41, 44]. The arterial phase is generally characterized by a rim enhancement; though it is possible, there will be no enhancement. i-CCA can also show a globally hyperenhanced pattern, mainly in patients with chronic hepatitis or cirrhosis [45]. The diagnosis of i-CCA requires the use of second-level imaging techniques, and biopsy using immunohistochemistry techniques should be performed for histologic examination [45–47] (Strong recommendations, low grade of evidence.) Strong consensus (35 approved/0 disapproved/0 abstained. 100%).

#### Liver metastasis

The ultrasound aspect of metastatic lesions of the liver is variable, and can include any ultrasound appearance. Although ultrasound examination is frequently requested at the first diagnosis and during follow-up in patients with oncological disease, the sensitivity of conventional ultrasound in the detection of liver metastases is lower than that of second-level imaging techniques (CEUS, CE CT and/or CE MRI). This is particularly true when lesions are < 1 cm. Color Doppler US is not indicated for the study of hepatic metastases. In CEUS, hepatic metastases are characterized by washout in the early portal phase [3]. The accuracy of CEUS in the detection of secondary liver lesions is superior to that of conventional ultrasound and comparable to that of CT and MRI, especially in easily explored patients [14, 20, 21, 48]. (Strong recommendations, high grade of evidence.) Strong consensus (35 approved/0 disapproved/0 abstained. 100%).

## List of Abbreviations

AASLD: American Association for the study of Liver Diseases
ACG: American College of Gastroenterology
AIOM: Italian Association of Medical Oncology
AISF: Italian Association for the study of the liver
CCC: Cholangiocellular carcinoma
CT: Computed tomography
CECT: Contrast-enhanced computed tomography
CEMRI: Contrast-enhanced magnetic resonance imaging
CEUS: Contrast-enhanced ultrasound
DAA: Directly active antiviral
EASL: European Association for the Study of the liver
EASL–EORTC: European Association for the Study of the Liver—European Organization for Research and Treatment of Cancer
EFSUMB: European Federation of Societies for Ultrasound in Medicine and Biology
FNH: Focal nodular hyperplasia
GRADE: Grading of Recommendations Assessment Development and Evaluation
HCA: Hepatocellular adenoma
HCC: Hepatocellular Carcinoma
UCA: Ultrasound contrast agent
US: Ultrasound or ultrasonography
SIC: Italian Society of Surgeon
SIRM: Italian Society of Medical and Interventional Radiology
SITO: Italian Society of Organ’s Transplantation
SIUMB: Italian Society of Ultrasound in Medicine and Biology
SNLG: National System of Guidelines
IT-IHPBA: International Hepato-Pancreato Biliary Association
WFUMB: World Federation for Ultrasound in Medicine and Biology
